# Does Increasing Treatment Frequency Address Suboptimal Responses to Ivermectin for the Control and Elimination of River Blindness?

**DOI:** 10.1093/cid/ciw144

**Published:** 2016-03-21

**Authors:** Kwadwo K. Frempong, Martin Walker, Robert A. Cheke, Edward Jenner Tetevi, Ernest Tawiah Gyan, Ebenezer O. Owusu, Michael D. Wilson, Daniel A. Boakye, Mark J. Taylor, Nana-Kwadwo Biritwum, Mike Osei-Atweneboana, María-Gloria Basáñez

**Affiliations:** 1 Noguchi Memorial Institute for Medical Research, University of Ghana, Legon; 2 London Centre for Neglected Tropical Disease Research, Department of Infectious Disease Epidemiology, School of Public Health, Imperial College London; 3 Natural Resources Institute, University of Greenwich at Medway, Chatham Maritime, United Kingdom; 4 Council for Scientific and Industrial Research, Water Research Institute, Accra; 5 Department of Animal Biology and Conservation Science, University of Ghana, Legon; 6 Department of Parasitology, Liverpool School of Tropical Medicine, United Kingdom; 7 Ghana Health Service, Accra

**Keywords:** onchocerciasis, ivermectin, biannual treatment, suboptimal responses, elimination

## Abstract

***Background.*** Several African countries have adopted a biannual ivermectin distribution strategy in some foci to control and eliminate onchocerciasis. In 2010, the Ghana Health Service started biannual distribution to combat transmission hotspots and suboptimal responses to treatment. We assessed the epidemiological impact of the first 3 years of this strategy and quantified responses to ivermectin over 2 consecutive rounds of treatment in 10 sentinel communities.

***Methods.*** We evaluated *Onchocerca volvulus* community microfilarial intensity and prevalence in persons aged ≥20 years before the first, second, and fifth (or sixth) biannual treatment rounds using skin snip data from 956 participants. We used longitudinal regression modeling to estimate rates of microfilarial repopulation of the skin in a cohort of 217 participants who were followed up over the first 2 rounds of biannual treatment.

***Results.*** Biannual treatment has had a positive impact, with substantial reductions in infection intensity after 4 or 5 rounds in most communities. We identified 3 communities—all having been previously recognized as responding suboptimally to ivermectin—with statistically significantly high microfilarial repopulation rates. We did not find any clear association between microfilarial repopulation rate and the number of years of prior intervention, coverage, or the community level of infection.

***Conclusions.*** The strategy of biannual ivermectin treatment in Ghana has reduced *O. volvulus* microfilarial intensity and prevalence, but suboptimal responses to treatment remain evident in a number of previously and consistently implicated communities. Whether increasing the frequency of treatment will be sufficient to meet the World Health Organization's 2020 elimination goals remains uncertain.

## Abstract (precis)

The first 3 years of biannual ivermectin distribution in Ghana have substantially reduced *Onchocerca volvulus* infection levels in 10 sentinel communities, but longitudinal analysis indicates that some communities are still consistently responding suboptimally to treatment, with implications for onchocerciasis elimination.

In 1987, soon after ivermectin became licensed for human use [1], and following the first community trials [2], Ghana became one of the first countries to introduce mass treatment to control onchocerciasis (river blindness). Ivermectin kills *Onchocerca volvulus* microfilariae (the larval progeny of adult worms that are transmissible to *Simulium* blackfly vectors) and temporarily sterilizes female worms such that numbers of microfilariae remain suppressed for at least 3 months following treatment [3]. Subsequently, females regain fertility and microfilariae repopulate the skin. Hence, ivermectin can only control onchocerciasis-associated disease—caused by immunopathological responses to chronic infection of the skin and ocular tissue by microfilariae [4]—when given at regular intervals. Infection can be eliminated if microfilariae are suppressed long enough to ensure that transmission is interrupted for at least 10 years, the average lifespan of adult worms [5]. Mass treatments with ivermectin have successfully eliminated onchocerciasis from foci in Mali and Senegal [6] (with annual or biannual distribution), Nigeria [7], Mexico [8], Colombia [9], Ecuador [10], and northern Venezuela [11]. (The strategy in Latin America has been mostly biannual treatment.) National programs in Ethiopia and Uganda, among others, have adopted biannual distribution in some foci to accelerate progress toward elimination [12–14].

Despite years of ivermectin treatment in Ghana, and vector control in its savannah habitats, onchocerciasis still affects thousands of communities within 66 districts [15], and approximately 3.2 million people remain at risk of infection [16]. The resilience of onchocerciasis is probably partly due to poor responses to ivermectin in several Ghanaian communities [17, 18], raising fears of decreased ivermectin efficacy. In a community of normally responding individuals, microfilariae are expected to reach about 10% of their pretreatment numbers 6 months after treatment, and about 20% one year after treatment [3, 17]. In suboptimally responding communities, microfilarial repopulation rates 6 months after treatment have been observed at >50% [19]. Some of these communities are those that have been treated with the most rounds of ivermectin [20].

In 2010, in response to the persistence of onchocerciasis in Ghana, the Neglected Tropical Diseases Programme (NTDP) of the Ghana Health Service (GHS) adopted a biannual treatment strategy in 44 of 77 endemic communities [21]. Here, we report microfilarial loads and prevalence in 10 NTDP sentinel communities—some previously identified as responding suboptimally to ivermectin—before and after 4 (or 5) rounds of biannual treatment. We evaluate responses to ivermectin by estimating rates of microfilarial repopulation in cohorts of individuals followed up at 3 and 6 months after treatment, comparing skin repopulation rates with community endemicity, therapeutic coverage, and number of years of prior ivermectin treatment. We discuss our results in the contexts of historical epidemiological data collected from these communities during annual ivermectin distribution and the World Health Organization's (WHO) goals to eliminate onchocerciasis [22].

## METHODS

### Ethical Approval

Ethical approval was obtained from the ethics review committees of the Noguchi Memorial Institute for Medical Research, Ghana (NMIMR-IRB CPN 032110-11), the Ghana Health Service (GHS ERC 04_3_11), and the Imperial College London Research and Ethics Committee (ICREC_11_2_4).

### Study Site

**Figure 1.**
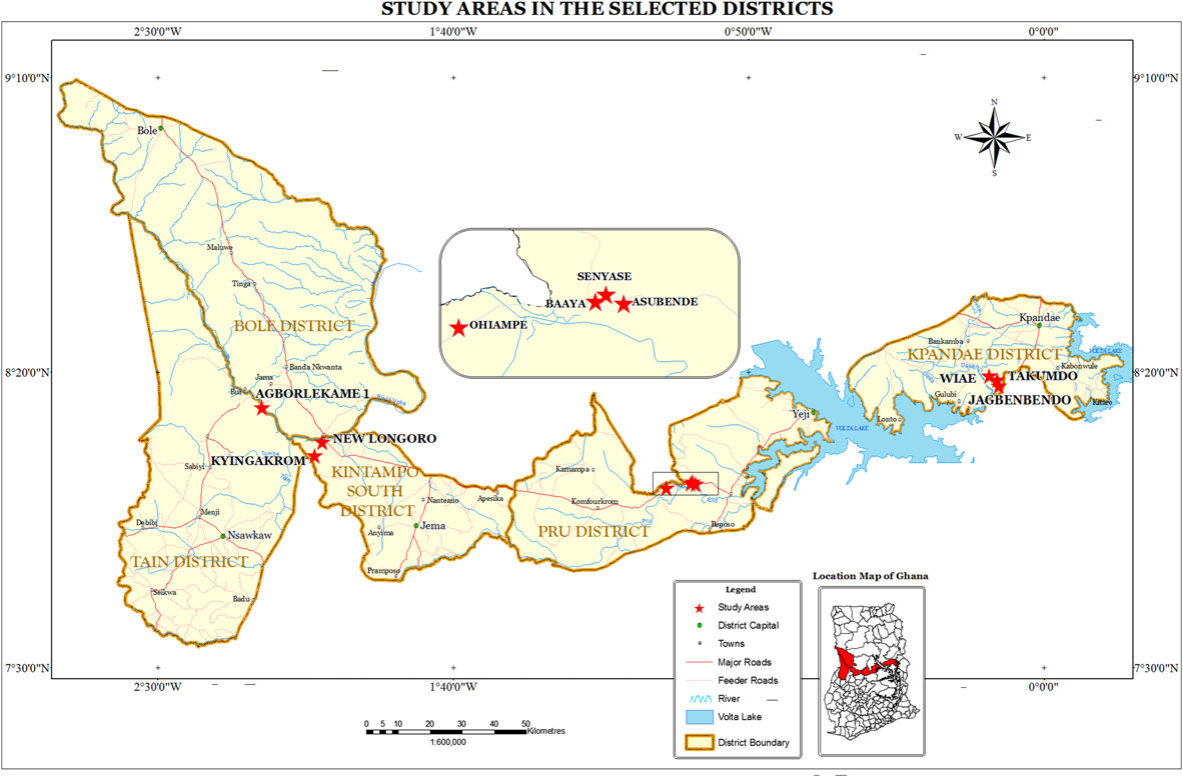
Map of Ghana indicating administrative regions and locations of study communities.

The study was conducted in 10 onchocerciasis-endemic communities within savannah regions of Ghana (Figure 1). The communities were selected from some of the endemic areas where concerns on ivermectin efficacy have been previously reported [19]. By the time of this investigation, study communities had received between 14 and 23 rounds of annual ivermectin treatment.

### Study Design

The 10 selected communities had been scheduled to receive mass biannual treatments with ivermectin from July 2010. We used the inclusion/exclusion criteria for selecting communities described elsewhere [19], including communities previously identified as responding suboptimally to ivermectin [17, 18, 20]. We recruited adults aged ≥18 years, randomly selected from different households. The number of eligible participants represented about 50%–70% of the total population within the 10 studied communities. Those who were included represented about 10%–40% of the total population and, of the total eligible population, approximately 70% in small communities (such as Asubende, with a population of 87) and roughly 20% in larger communities (such as New Longoro and Wiae with populations of 1650 and 1611, respectively). The objectives and schedules of the study were explained to every individual, and those who agreed to participate signed a consent form.

**Figure 2.**
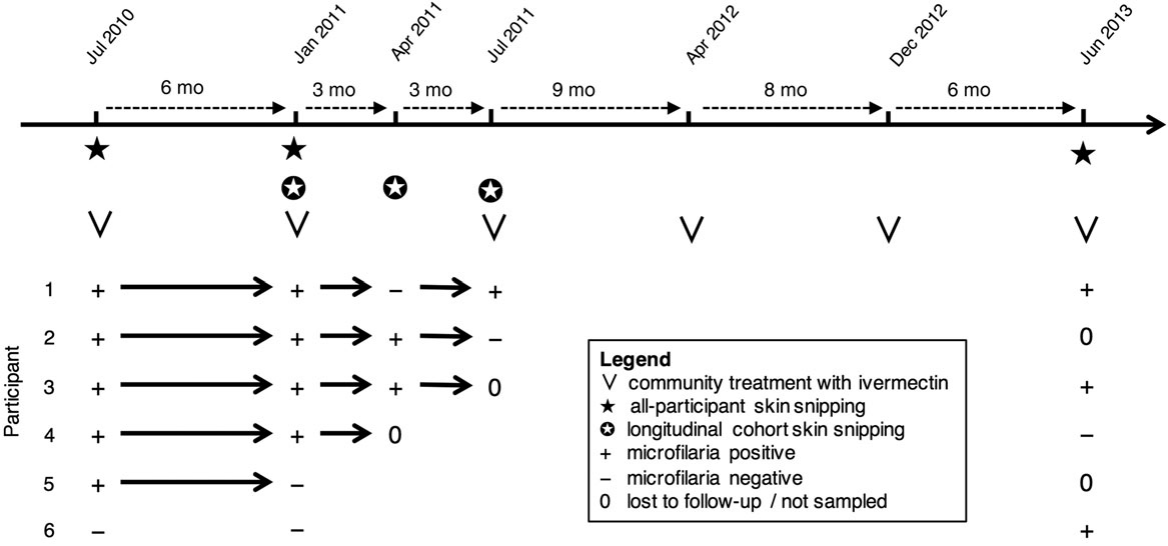
Schematic timeline and illustrative history of 6 trial participants of the study used to evaluate community trends in infection and rates of microfilarial repopulation following the onset of a biannual treatment strategy in 10 Ghanaian communities. Participants 1–6 represent 6 of the 956 consenting individuals from whom skin snips were taken in July 2010, just before the first round of biannual ivermectin treatment, and 6 months later in January 2011, just before the second round of biannual ivermectin treatment. Participants 1–5 were positive for microfilariae in July 2010 and hence were included in the cohort of 217 individuals for evaluating rates of skin microfilarial repopulation. Participants 1–4 represent 4 of the 186 individuals who were microfilaria positive in January 2011, with participants 1 and 2 successfully followed up and skin snipped in April 2011 and again in July 2011, just before the third round of biannual ivermectin treatment. Participants 1, 3, 4, and 6 represent 4 of the original 956 participants who agreed to be skin snipped for a final time in June 2013, just before the final round of treatments delivered by the Ghana Health Service Neglected Tropical Diseases Programme. The months given on the timeline are the modal months of treatment activity among the 10 communities, but there is significant variation in the months and exact dates, especially for the biannual treatments given after July 2011 (see Figure 3 for exact dates).

Figure 2 illustrates the study design and times of treatment with ivermectin (150 µg/kg, directly observed) using an example timeline of 6 trial participants. Skin snips of 956 consenting participants were taken in July 2010 just before the first round of biannual ivermectin treatment. All 956 participants were skin snipped 6 months later, in January 2011, just before the second biannual treatment round. A total of 217 (22.7%) of these participants (Table 1), who were positive for microfilariae in July 2010 (eg, participants 1–5 in Figure 2), formed a cohort for evaluating rates of skin microfilarial repopulation. Within this cohort, the 186 participants (Table 1) positive for microfilariae in January 2011 (eg, participants 1–4 in Figure 2) were skin snipped in April 2011 and in July 2011, just before the third round of biannual ivermectin treatment (some participants were lost to follow-up, eg, participants 3 and 4 in Figure 2). Three additional rounds of ivermectin treatment were distributed approximately every 6 months, in April 2012, December 2012, and June 2013, as part of GHS NTDP activities. Before the final round of treatment, in June 2013, a final round of skin snipping of consenting participants (eg, participants 1, 3, 4, and 6 in Figure 2) was repeated. Techniques used to count microfilariae in skin snip biopsies are described in the Supplementary Methods.

**Table 1.** Longitudinal Cohorts of Participants in 10 Ghanaian Communities Who Were Followed up and Skin Snipped Over the First 2 Rounds of Biannual Treatment With Ivermectin

Community	Month and Year (Months Since Preceding Round of Treatment)			
	July 2010 (0)^a^	January 2011 (6)^b^	April 2011 (3)	July 2011 (6)
Agborlekame 1	63	27	23	20
Asubende	34	9	8	9
Baaya	129	1	1	1
Jagbenbendo	107	50	47	46
Kyingakrom	82	14	12	12
New Longoro	126	17	13	15
Ohiampe	85	5	5	4
Senyase	64	8	6	7
Takumdo	108	50	48	44
Wiae	158	26	23	24
Total	956	217	186	182

^a^ Only participants positive for microfilariae were followed up in January 2011.

^b^ Only participants positive for microfilariae were followed up in April 2011 and July 2011.

### Community Microfilarial Load and Community Microfilarial Prevalence

We calculated community microfilarial load (CMFL) [23] and community microfilarial prevalence (CMFP) as our primary and secondary indicators of community infection levels in the adult (aged ≥20 years) population. These were calculated before the first round of biannual treatment in July 2010, before the second round in January 2011, and before the fifth or sixth round in March 2013 or June 2013 (the schedules of each community differed slightly). CMFL and CMFP calculations are given in the Supplementary Methods.

### Community Treatment History and Coverage

We obtained data on community treatment coverage (Supplementary Figure A) at all treatment rounds from the NTDP to facilitate interpretation of CMFL and CMFP throughout the study. Coverage was calculated using treatment and census data provided by the community ivermectin distributors to the NTDP. It refers to the therapeutic coverage in the total population. Historical records of coverage were also obtained from the GHS NTDP.

### Microfilarial Repopulation

We constructed log-linear marginal regression models [24] to describe the average number of microfilariae per skin snip (mf/ss) in the longitudinal cohort of 217 individuals (Table 1; Figure 2), adjusting for community, participant age, and sex. We constructed 2 models to analyze the data (Table 2). Both permit repopulation rates to vary among communities, but the first (Model 1A and 1B, Table 2) permits repopulation rates to vary between the 2 consecutive repopulation periods, whereas the second (Model 2, Table 2) estimates a single community-specific repopulation rate, combining information from both repopulation periods. Mathematical details are given in the Supplementary Methods.

**Table 2.** Key Features of the Log-Linear Marginal Regression Models Used to Describe the Observed Microfilarial Counts in the Longitudinal Cohort

Type	Variant	Key Features
Model 1	A and B	Response/outcome variable defined by individual microfilarial counts Modeled mean number of microfilariae per participant adjusted for the covariates age group (18–20, 21–40, 41–60, and 61–80), sex, and community Microfilarial repopulation rates permitted to vary among communities and between repopulation periods by including sampling time as a categorical covariate interacting with community
	B	Microfilarial repopulation rates adjusted by exact number of days since preceding round of ivermectin treatment yielding standardized repopulation rates (eg, 6-month repopulation rates)
Model 2	…	Response/outcome variable defined by individual microfilarial counts Modeled mean number of microfilariae per participant adjusted for the covariates age group (18–20, 21–40, 41–60, and 61–80), sex, and community A single microfilarial repopulation rate estimated for each community, combining information from both repopulation periods, by including sampling time as a continuous covariate—defined as days since preceding ivermectin treatment—interacting with community Additive, community-wide adjustments for potentially different repopulation rates between 2 repopulation periods

We compared microfilarial repopulation rates graphically and by identifying communities with statistically significantly different estimates compared with a reference community (Takumdo). We also explored graphically how repopulation rates correlated with prior number of years of ivermectin treatment, therapeutic coverage, and CMFL just before the start of biannual treatment.

## RESULTS

### Trends in Community Infection

**Figure 3.**
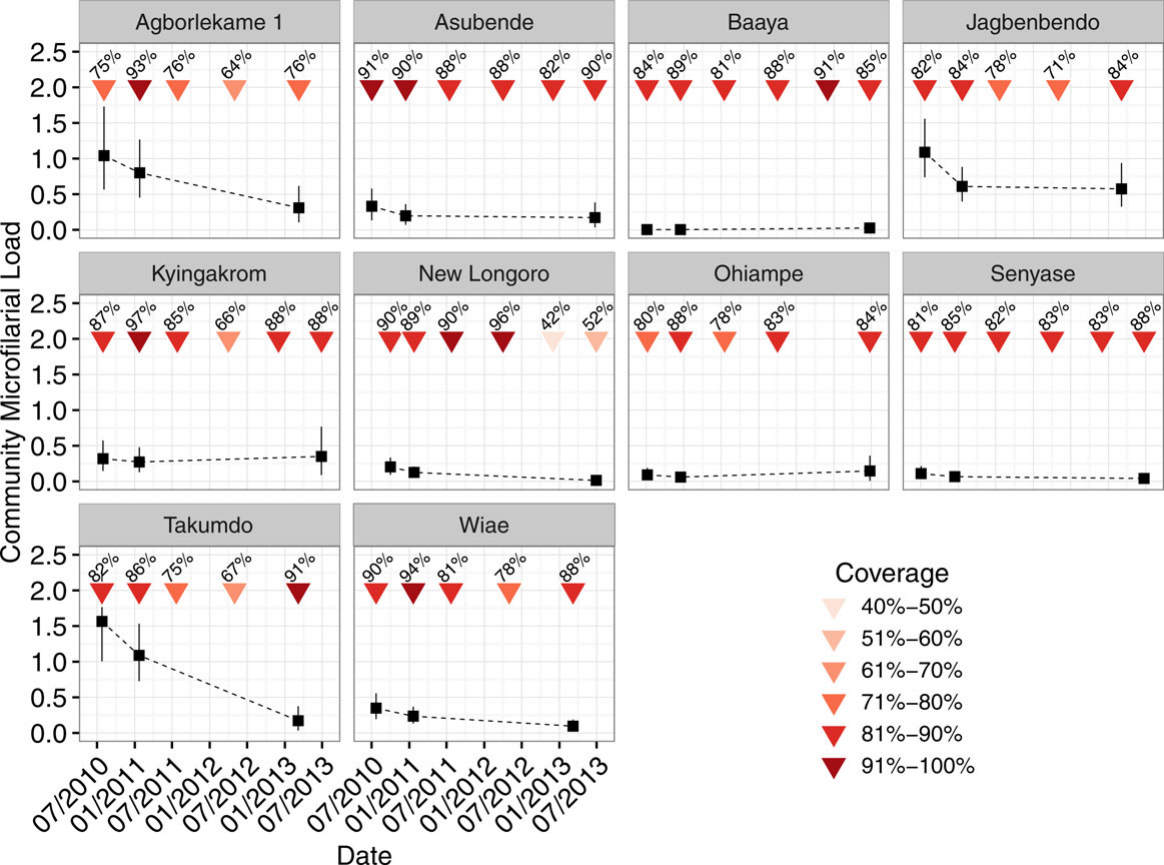
Trends in community microfilarial loads (CMFLs) in 10 Ghanaian communities from the onset of a biannual ivermectin treatment strategy. CMFL is defined as the geometric mean number of microfilariae per skin snip in people aged ≥20 years. Colored arrows indicate dates when mass treatment with ivermectin was distributed, by either the authors or the community ivermectin distributors. Ivermectin was administered directly after skin snipping on dates when microfilarial load was assessed. Data on the community therapeutic coverage of ivermectin were collated by the Ghana Health Service. The 6 scheduled rounds of biannual ivermectin treatment were successfully delivered to only 5 (Asubende, Baaya, Kyingakrom, New Longoro, and Senyase) of the 10 communities; the other communities (Agborlekame 1, Jagbenbendo, Ohiampe, Takumdo, and Wiae) achieved 5 rounds of biannual treatment. Vertical lines indicate 95% confidence intervals calculated using a nonparametric bootstrap technique (Supplementary Methods). Dotted lines join estimated values and are for presentation purposes only. Triangles indicate times of ivermectin treatment, and numbers above triangles indicate the therapeutic coverage in the whole community.

Figure 3 presents community-specific CMFLs calculated in July 2010, January 2011, and March (or June) 2013 (CMFPs are presented in Supplementary Figure B). We include the dates when each round of biannual treatment was distributed and the population coverage. The impact of the first round of biannual treatment appears somewhat greater than that in subsequent rounds, as demonstrated by the slightly faster decline in CMFL between round 1 (July 2010), and round 2 (January 2011), compared with that between round 2 and the final assessment of infection levels in March (or June) 2013 (compare the gradients of the dotted lines in Figure 3). This trend is most apparent in Asubende, Jagbenbendo, New Longoro, Senyase, and Wiae, and least pronounced in Agborlekame 1 and Takumdo. In Ohiampe, community infection levels were greater in June 2013 than in July 2010, despite 4 rounds of treatment (1 round was missed in the first quarter of 2013).

### Trends in Microfilarial Repopulation

**Figure 4.**
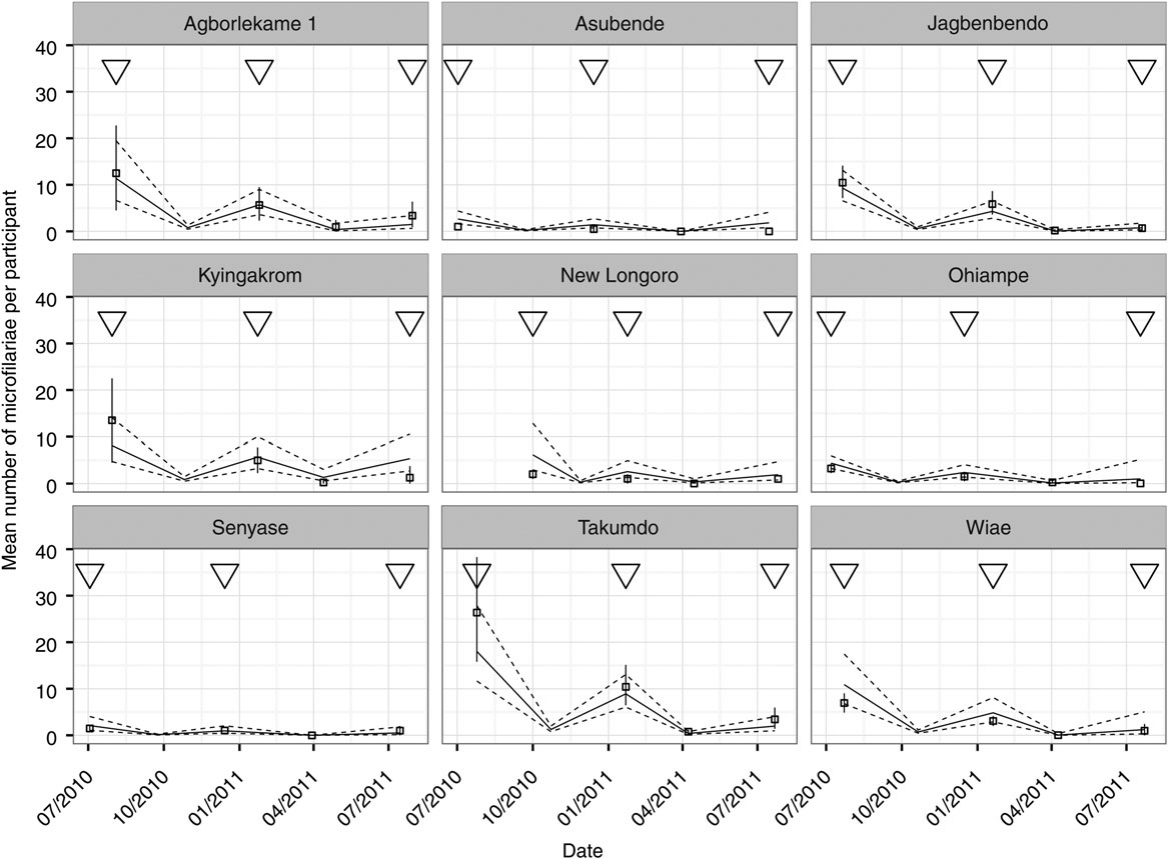
Trends in mean numbers of microfilariae per participant in 10 Ghanaian communities from the onset of a biannual ivermectin treatment strategy. Data points represent observed mean microfilarial loads, by community, in the reference strata of males within the age group 21–40 years. The solid vertical lines are corresponding 95% bootstrap confidence intervals. Triangles indicate when ivermectin was administered to the study participants. Solid lines join fitted estimated values—and in the case of October 2010, predicted values—from the marginal regression model that includes additive stratum adjustments for age group and sex, and interactive adjustments between sampling date and community (Model 1A in Table 2). Dotted lines join the corresponding 95% confidence bounds calculated using robust sandwich estimators of coefficient standard errors (Supplementary Methods). The predicted values in October 2010 are provided to assist the reader to envisage the likely dynamics in mean numbers of microfilariae per skin snip between the first and second sampling dates. These predictions were generated from the marginal regression model that treats the time since the preceding ivermectin treatment as a continuous variable (Model 2 in Table 2) and assumes that (hypothetical) microfilarial sampling took place midway between the July 2010 and January 2011 sampling times. Data from Baaya are not shown because only 1 participant was microfilaria positive and followed up in this community (Table 1), leading to very large associated estimates of uncertainty.

Figure 4 presents the observed and model-fitted (Model 1A, Table 2) mean number of mf/ss by sampling date and community in the reference demographic stratum of males in the age group 21–40 years. We also include the model-predicted mean number of mf/ss in October 2010 (3 months after the first round of biannual treatment), indicating the likely microfilarial dynamics during the first 6-month repopulation period. In general, mean numbers of mf/ss per stratum are lower after the second repopulation period than after the first; microfilariae cannot repopulate completely in 6 months before further suppression by another treatment round. Mean numbers of mf/ss per stratum in January 2011, 6 months after the start of biannual treatment, are quite high compared with those in July 2010 (one expects microfilariae to reach about 10% of their pretreatment population level after 6 months [3]).

### Microfilarial Repopulation Rates

**Figure 5.**
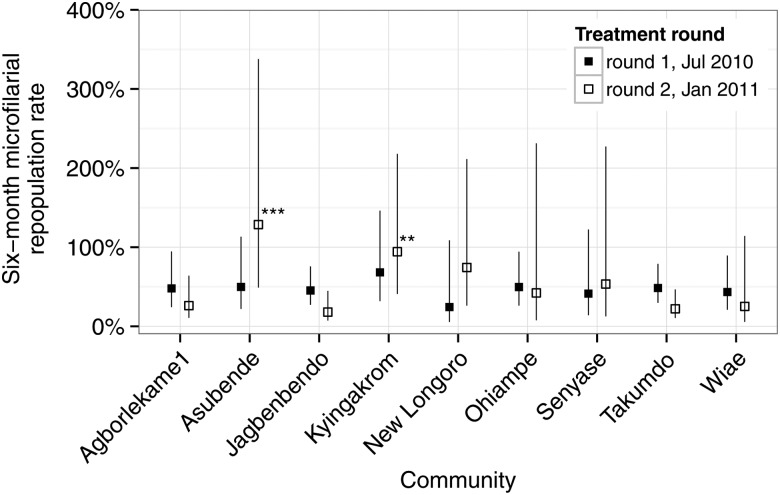
Six-month microfilarial repopulation rates in 10 Ghanaian communities from the onset of a biannual ivermectin treatment strategy. Filled and open data points represent, respectively, estimated mean microfilarial loads 6 months after the first or second round of ivermectin treatment, expressed as a percentage of the microfilarial load estimated just before the preceding round of ivermectin treatment. These estimates are derived from Model 1B, Table 2, which adjusts for the variable follow-up times among communities, permitting estimation of directly comparable standardized 6-month rates of repopulation. The 6-month microfilarial repopulation rate from Baaya is not shown because only 1 participant was microfilaria positive and followed up in this community (Table 1), leading to very large associated estimates of uncertainty. Vertical lines indicate 95% confidence bounds, calculated using robust sandwich estimators of coefficient standard errors (Supplementary Methods). *P* values comparing the rate of repopulation with the reference village of Takumdo: ***P < .001; **P < .01.

We define the rate of microfilarial repopulation as the mean number of mf/ss expressed as a percentage of the mean immediately before the preceding treatment with ivermectin. This captures how quickly microfilariae reappear in the skin between consecutive treatment rounds. Figure 5 provides standardized 6-month repopulation rates, adjusted by the differing exact durations between sampling times (calculated from Model 1B in Table 2; nonstandardized repopulation rates are depicted in Supplementary Figure C). These estimates confirm that microfilarial repopulation rates are generally quite high—typically approximately 50% during the first period of repopulation—and similar, albeit somewhat more variable, after the second repopulation period. The repopulation rates in Asubende and Kyingakrom after the second round of treatment are statistically significantly higher than in the reference community of Takumdo.

**Figure 6.**
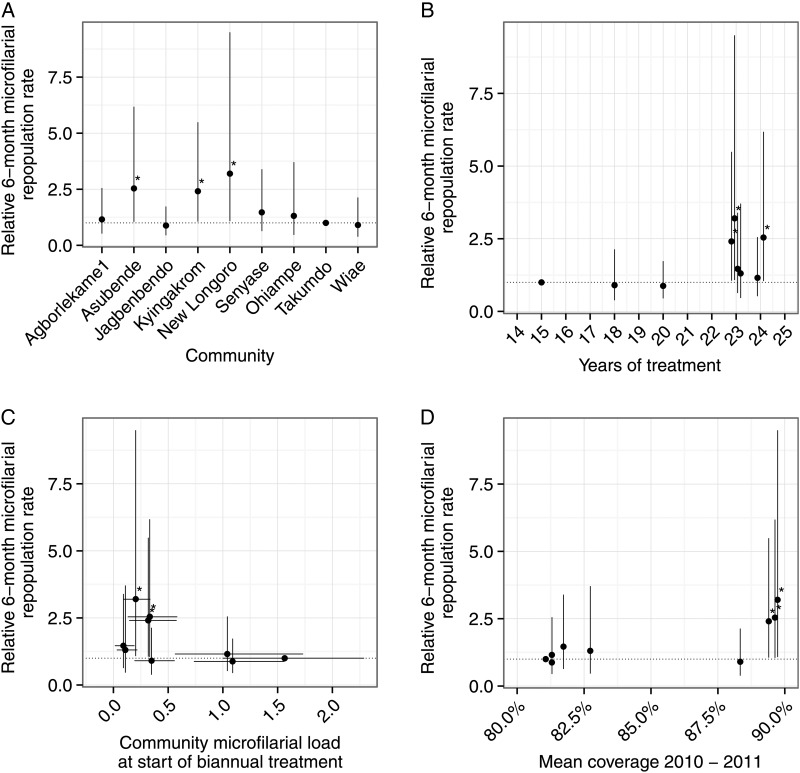
Relative 6-month microfilarial repopulation rates in 10 Ghanaian communities over the first 2 rounds of a biannual ivermectin treatment strategy. Data points represent the estimated relative (multiplicative) 6-month microfilarial repopulation in each community compared with Takumdo. Six-month repopulation rates are defined as mean microfilarial loads 6 months after a round of ivermectin treatment, expressed as a percentage of the microfilarial load estimated just before the preceding treatment round. Estimates are derived from Model 2, Table 2, which treats time since the preceding ivermectin treatment as a continuous covariate interacting with the indicator covariate for community. The 6-month microfilarial repopulation rate from Baaya is not shown because only 1 participant was microfilaria positive (Table 1), leading to very large associated estimates of uncertainty. *A*, Estimates are plotted side-by-side for the different communities. *B*, Estimates are plotted against the number of years of ivermectin treatment preceding the biannual strategy. *C*, Estimates are plotted against community microfilarial load (CMFL) preceding the first biannual ivermectin treatment. *D*, Estimates are plotted against the mean coverage of ivermectin distribution for the years 2010 and 2011, corresponding to the component of the study when the longitudinal cohort of participants was followed up over 2 consecutive rounds of biannual treatment (see also Supplementary Figure A for disaggregated coverage data from 2005 to 2013). Vertical lines are 95% confidence bounds, calculated using robust sandwich estimators of coefficient standard errors (Supplementary Methods). Solid horizontal lines in (*C*) indicate 95% confidence bounds associated with the estimated CMFL, calculated using a numerical bootstrap resampling method (Supplementary Methods). *P < .05, comparing with the reference village of Takumdo.

Figure 6 presents the single relative rates of repopulation by community (estimated using Model 2, Table 2) compared to Takumdo. Over both repopulation periods, rates of repopulation are statistically significantly (*P* < .05) higher in Asubende, Kyingarom, and New Longoro compared with Takumdo. Graphically, we find no obvious association between the relative rate of microfilarial repopulation and (1) the number of annual treatments with ivermectin before the start of the study (Figure 6*B*), (2) the CMFL before the first biannual treatment (Figure 6*C*), or (3) the average coverage of ivermectin distribution during the cohort component of the study (Figure 6*D*; see Supplementary Figure A for disaggregated coverage data).

## DISCUSSION

Onchocerciasis in Ghana remains resilient to the long-standing and large-scale (antivectorial and antiparasitic) interventions implemented over the past 40 years [25]. Despite having been earmarked for elimination as a public health problem by 2015 [16], there exist persistent hotspots of transmission [19, 26, 27] and reports of *O. volvulus* microfilariae repopulating the skin of patients faster than expected following treatment with ivermectin [19, 20], a phenomenon also documented in Cameroon [28]. In 2010, and responding to this challenge, the GHS implemented biannual mass ivermectin treatment in many endemic communities [21]. We report on trends in community-wide infection with *O. volvulus* in 10 Ghanaian communities over the first 3 years of this biannual strategy and evaluate rates of microfilarial repopulation in cohorts of participants over the first 2 rounds of treatment.

The last systematic evaluation of community infection levels in many of the studied communities was in 2004–2005, after 10–18 annual mass ivermectin treatments [19] (Supplementary Tables A and B). Comparing these values with infection levels in July 2010 shows that the intervening 6 years of annual ivermectin mass treatment have reduced CMFLs generally by at least 50%. Infection levels were further reduced by March or June 2013, after 3 years of biannual treatment. Reductions in CMFL were >36% in most communities and the CMFP was statistically significantly <10% in 5 of 10 communities (Supplementary Tables A and B). Hence, the biannual strategy has had a positive impact.

Whether residual infection levels constitute a public health problem would be best evaluated by measuring levels of onchocerciasis-associated morbidity. However, it is hard to envisage declaring the problem eliminated in communities where microfilarial prevalence is >10%, or >20% as in Jagbenbendo. Moreover, whether biannual treatments will ultimately be sufficient to eliminate infection will depend on local transmission and programmatic conditions, particularly on the intensity of blackfly biting [25, 26] and the sustainability of high levels of treatment coverage and adherence [29, 30]. One of the objectives of the Neglected Tropical Diseases Modelling Consortium (www.ntdmodelling.org) is to determine what intervention strategies will be necessary to eliminate infection in the timelines set out by the WHO Roadmap on Neglected Tropical Diseases [22].

The 6-month rates of repopulation estimated here are broadly around 50% and are high compared with the expected 10% from parasite populations predominantly naive to ivermectin [3]. They are also higher than those estimated from some of the same communities in 2005, which were typically <30% (Supplementary Table C). Some of this discrepancy is probably because the 10% value (and the previous estimates from these communities) was based on geometric means, which are not strictly comparable with the model-derived repopulation rates presented here (which correspond to arithmetic means). Furthermore, the sampling scheme employed in this study (and previously in the same communities [19]) followed up only participants who were positive for microfilariae at recruitment. This ensures that only people infected with *O. volvulus* are repeatedly skin snipped, increasing the efficiency of sampling when the prevalence of infection is low. Unfortunately, this necessary protocol potentially introduces sampling biases because the sensitivity of skin snipping declines with decreasing infection intensity [31]. Hence, participants with less intense infections are more likely to be erroneously deemed uninfected and not followed up. This will probably upwardly bias repopulation rates because more intensely infected people will have more microfilariae after a period of repopulation than those with less intense infections.

Notwithstanding these cautions, the 3 communities with the highest repopulation rates over the 2 repopulation periods (Asubende, Kyingakrom, New Longoro) have been previously implicated as responding suboptimally to ivermectin [19, 20, 27]. A mechanistic cause underlying these observations cannot be determined from the statistical analysis presented here. However, previous suggestions that faster rates of skin repopulation by microfilariae might result from a sudden increase in new infections between treatment rounds—perhaps due to programmatic deficiencies in coverage and compliance [32, 33]—are difficult to reconcile with the generally high levels of therapeutic coverage observed throughout (Figure 6*D*) and before (Supplementary Figure A) the study. It is more likely that transmission has been declining since the onset of biannual ivermectin treatment in July 2010, as evidenced by the generally falling CMFL (Figure 3), although the resilience of community infection levels to biannual distribution in Kyingakrom is noteworthy (Supplementary Tables A and B).

Work is ongoing to evaluate the genotype of adult parasites extracted from some of the participants of this study. Previous analyses comparing allele frequencies among adult female *O. volvulus* infecting people in multiply treated and ivermectin-naive populations in Ghana and Cameroon identified selection of P-glycoprotein and β-tubulin genes, both associated with resistance to ivermectin in helminth infections of livestock [34, 35]. Moreover, a genetic analysis of the entire region of the β-tubulin gene extracted from worms infecting people from Kyingakrom—a consistently implicated suboptimally responding community—has identified statistically significantly higher frequencies of 6 single-nucleotide polymorphisms [36]. How the phenotypic response of individual worms relates to these genetic changes remains incompletely understood. Worms collected from suboptimally responding communities have been associated with higher fertility than worms from putatively normally responding communities [36], possibly indicative of a faster resumption of fertility following exposure to ivermectin [28]. However, results elsewhere suggest that selection driven by exposure to ivermectin is associated with a pleiotropic fitness cost of decreased fertility [35], so perhaps putatively resistant worms can resume production of microfilariae more rapidly than their susceptible counterparts, but ultimately have less reproductive potential.

Our conclusions on microfilarial repopulation rates are based on average, community estimates, adjusted for individual (host) characteristics such as age and sex. This is consistent with the inferential basis of previous, more descriptive analyses of data from some of the same communities studied here [19, 27]. Yet, particularly for these well-studied and relatively small communities, many of the same individuals have probably repeatedly participated in the epidemiological studies undertaken over the last 15 years. Hence, future analyses should focus on estimating drug responses at the individual level [37, 38]. It is more plausible that certain individuals, rather than entire communities, are consistently responding poorly to ivermectin (and influencing the community-wide response). Poor individual responses to treatment might be caused by host-related factors or, given the long lifespan of adult *O. volvulus*, by drug-tolerant parasites.

The biannual ivermectin treatment strategy is markedly reducing *O. volvulus* infection levels in Ghana. However, despite high and sustained therapeutic coverage, suboptimal responses to ivermectin persist in previously implicated communities. Whether this is caused by drug-tolerant or resistant parasites, or by host-related factors, remains unclear. Analyses are yet to be performed to test the hypothesis that community-level suboptimal responses are driven by a minority of consistently poorly responding individuals (or their worms) and to identify underlying mechanisms. The EPIONCHO and ONCHOSIM mathematical transmission models are being used to assess the feasibility of meeting the WHO elimination goals with annual or biannual ivermectin treatment [29, 30, 38, 39], and in the future they will be used to establish which settings may require alternative or complementary strategies (such as test-and-treat macrofilaricidal doxycycline therapy [40] and/or focal vector control). Such modeling projections cover a wide range of epidemiological and programmatic contexts, but should also accommodate the possibility that ivermectin may not be as universally efficacious as hoped.

## Supplementary Data

Supplementary materials are available at http://cid.oxfordjournals.org. Consisting of data provided by the author to benefit the reader, the posted materials are not copyedited and are the sole responsibility of the author, so questions or comments should be addressed to the author.

Supplementary Data
