# Supplementary material for: Does Increasing Treatment Frequency Address Suboptimal Responses to Ivermectin for the Control and Elimination of River Blindness?
Source: Clin Infect Dis. 2016 Mar 21;62(11):1338–47. doi: 10.1093/cid/ciw144 (PMC4872292; doi:10.1093/cid/ciw144)
Supplement: Supplementary Data [file supp_62_11_1338__index.html]

Does Increasing Treatment Frequency Address Suboptimal Responses to Ivermectin for the Control and Elimination of River Blindness? — Supplementary Data 

# Does Increasing Treatment Frequency Address Suboptimal Responses to Ivermectin for the Control and Elimination of River Blindness?

## Supplementary Data

Supplementary Data

- Supplementary Data - pdf file
