# Supplementary material for: Does Increasing Treatment Frequency Address Suboptimal Responses to Ivermectin for the Control and Elimination of River Blindness?
Source: Clin Infect Dis. 2016 Mar 21;62(11):1338–47. doi: 10.1093/cid/ciw144 (PMC4872292; doi:10.1093/cid/ciw144)
Supplement: Supplementary Data [file supp_ciw144_ciw144Supp_Data.pdf]

Supplementary File

Does Increasing Treatment Frequency Address Sub-optimal Responses to Ivermectin for the Control and Elimination of River Blindness?

Kwadwo K. Frempong[1†], Martin Walker[2†], Robert A. Cheke[2, 3], Edward Jenner Tetevi[4], Ernest Tawiah Gyan[4], Ebenezer O. Owusu[5], Michael D. Wilson[1], Daniel A. Boakye[1], Mark J. Taylor[6], Nana-Kwadwo Biritwum[7], Mike Osei-Atweneboana[4], María-Gloria Basáñez[2]

[1]Noguchi Memorial Institute for Medical Research, University of Ghana, Legon, Ghana

[2] London Centre for Neglected Tropical Disease Research (LCNTDR), Department of Infectious Disease Epidemiology, School of Public Health, Imperial College London, UK

[3]Natural Resources Institute, University of Greenwich at Medway, Chatham Maritime, UK

[4]Council for Scientific and Industrial Research-Water Research Institute, Accra, Ghana

[5]Department of Animal Biology & Conservation Science, University of Ghana, Legon, Ghana

[6] Department of Parasitology, Liverpool School of Tropical Medicine, Liverpool, UK

[7]Ghana Health Service, Accra, Ghana

[†]Contributed equally to this work

Correspondence to:
Prof María-Gloria Basáñez
Department of Infectious Disease Epidemiology
Imperial College London
St Mary's campus
Norfolk Place
London, W2 1PG, UK
Office: +44 (0)20 7594 3295
m.basanez@imperial.ac.uk

Alternative contact:
Dr Mike Osei-Atweneboana
Department of Environmental Biology and Health
Council for Scientific and Industrial Research
Water Research Institute
P.O. Box M 32
Accra, Ghana
Office: +233 (0)21 777651, ext. 1002
oseiatweneboana@yahoo.co.uk

Supplementary Methods

Parasitological methods

At each sampling occasion (see Figure 2 main text), two skin snips were taken from the areas around the left and right iliac crests of each participant using a 2mm Holth-type corneoscleral punch. Samples were placed in a 0.9% sterile saline (or physiological saline) solution on 96-well microtitration plates and, using an inverted microscope, numbers of microfilariae were counted after 30 minutes and again after 24 hours of incubation. That is, unlike the protocol used by the Onchocerciasis Control Programme in West Africa (OCP) [1], biopsies that were positive for microfilariae at 30 minutes were not discarded but, along with those negative at 30 minutes, were incubated for 24 hours. This ensured that there was no systematic underestimation in the number of microfilariae counted from biopsies that were already positive for microfilariae at 30 minutes.

Community Microfilarial Load and Community Microfilarial Prevalence

The Community Microfilarial Load (CMFL) is the geometric mean number of microfilariae (including zero counts) per skin snip in people aged 20 years and above and was the reference index used by the OCP to assess the intensity of *Onchocerca volvulus* infection [2]. To mirror this intensity metric, we defined the Community Microfilarial Prevalence (CMFP) as the prevalence of microfilaridermia (microfilariae in the skin) in people aged 20 years and above (but note that it does not employ a transformation of the raw data as the CMFL does).

We calculated the CMFL for each community by first taking the arithmetic mean of the two microfilarial counts (from the right and left iliac crest snips) recorded per individual i, denoted m_i, adding 1 to each of these values, and then taking their geometric mean across individuals aged ≥20 years, subtracting 1 from such mean,

$$\text{CMFL} = \exp\left[\frac{\sum_{i=1}^{i=n} \ln(m_i + 1)}{n}\right] - 1, \qquad (1)$$

where n is the number of skin-snipped (≥ 20 year old) individuals within a community at a specific sampling time.

We calculated the CMFP for each community by first converting the two microfilarial counts per individual i into a binary variable of infected or not infected (where infected represents being positive for microfilariae in either or both snips), denoted p_i, and then taking the arithmetic mean of these values,

$$\text{CMFP} = \frac{\sum_{i=1}^{i=n} p_i}{n}. \qquad (2)$$

We used an ordinary non-parametric bootstrap re-sampling technique [3] to calculate 95% confidence intervals (CIs) associated with the value of the CMFL and CMFP for each community. This process involves:

(i) Re-sampling (at random) with replacement n participant microfilarial loads, m_i or binary indicators of infection, p_i.

(ii) Recalculating the CMFL and CMFP from the new sample using Equation (1) and Equation (2).

(iii) Repeating steps (i) and (ii) 10,000 times to yield an empirical sampling distribution of CMFL and CMFP.

(iv) Calculating the CIs from the 2.5% and 97.5% percentiles of the CMFL and CMFP sampling distributions.

Marginal regression models

Marginal models are a class of regression models that are suitable for analysing correlated data; in this case, correlation among repeated microfilarial counts measured from the same person. In particular, marginal models can be used in conjunction with robust, so-called sandwich estimators of coefficient standard errors [4] that, in turn, are used to calculate suitably adjusted (inflated) confidence intervals. (The thus inflated confidence intervals account for the correlation structure of the data and minimise Type I error in statistical

inference.) We defined two types of regression model (Model 1 and Model 2 in Table 2 of the main text) that were fitted to data on the microfilarial counts measured from the longitudinal cohort followed up from the initial sampling dates in July 2010 to six months after the second biannual treatment in July 2011.

For Model 1, we defined the sampling time as a categorical variable, interacting with the indicator variable of community, allowing repopulation rates to vary among communities. The model was constructed such that the mean number of microfilariae at a particular sampling time could be expressed as a percentage of the mean immediately before the preceding round of ivermectin treatment (i.e. either in July 2010 or January 2011, see Figure 2 of main text) by taking the exponent (because the models are log-linear) of the sum of the relevant covariate coefficients. We defined two variants of Model 1: (i) Model 1A, which ignores the exact number of days since the preceding round of ivermectin treatment and (ii) Model 1B, which incorporates the exact number of days since the preceding round of ivermectin treatment as an offset associated with the relevant sampling time (see Table 2 in the main text). By adjusting for the exact number of days since the preceding treatment, we were able to estimate directly comparable six-monthly repopulation rates for each community, one for each of the two consecutive periods of microfilarial repopulation (July 2010 to January 2011 and January 2011 to July 2011).

For Model 2, we defined the time since the preceding ivermectin treatment as a continuous variable interacting with the indicator variable for community. Like Model 1, this permitted rates of repopulation to vary among communities, but unlike Model 1, a single community-specific rate of microfilarial repopulation was estimated, informed by the data collected during both repopulation periods. Within this construct, we also incorporated treatment round as a categorical variable interacting with the time since the preceding treatment. This captures non community-specific heterogeneity in microfilarial repopulation rates between the consecutive repopulation periods (i.e. variation in repopulation rates between repopulation periods that affects all communities approximately equally). Such variation may arise, for example, from cumulative effects on the fertility of *O. volvulus* after repeated frequent exposures to ivermectin [5].

Mathematically, both Model 1 and Model 2 have a similar structure, with a systematic component specified as,

$$g(m_{ij}) = \mathbf{x}_{ij}\boldsymbol{\beta} \tag{3}$$

where m_{ij} is the expected value of microfilarial count j (for $j = 1,2$) from individual i (for $i = 1,2,...,n$); $\mathbf{x}_{ij}$ is a vector (collection) of covariates, and $\boldsymbol{\beta}$ is an accompanying vector of regression coefficients. The covariates within $\mathbf{x}_{ij}$ include a categorical variable indicating the time when a microfilarial count was measured (i.e. at day 0, 90 or 180 after ivermectin treatment); the community from which a count was measured; the additive covariates of age group [0,20], (20,40], (40,60] and (60,82] and sex, and the interactions between time and community. The inclusion of additive stratum adjustments for age and sex means that the mean (expected) number of microfilariae can vary among these strata. The interactions between sampling date and community ensures that rates of repopulation can vary among communities, but are constant within community strata. The difference between Model 1 and Model 2 arises in the specific construction of the interaction term, namely, in Model 1, time is a categorical variable excluding (Model 1A) or including (Model 1B) an offset for the exact number of days since the preceding round of ivermectin treatment; in Model 2, time is a continuous variable indicating the exact number of days since the preceding round of ivermectin treatment. Since the longitudinal cohort was followed up at only 3 and 6 months after the preceding ivermectin treatment, the underlying assumption of Model 2 (with time as a continuous covariate) is that numbers of microfilariae between these times (3 and 6 months) increase approximately log-linearly. This assumption appears to capture adequately the trend in the data, as shown in Figure 6 in the main text.

The variance of both model types, $v(m_{ij})$, was specified as a linear function of the mean, permitting extra-Poisson variation (overdispersion),

$$v(m_{ij}) = \varphi\, m_{ij}, \tag{4}$$

where φ is an estimated scale parameter. The correlation structure of the repeated measures was assumed to be exchangeable among microfilarial count measures from the same participant at the same time. That is, a single correlation parameter, ρ_1, was used to define the 'cross-sectional' correlation among repeated measures made at day 0—before treatment—or at days 90 or 180—after treatment. The correlation among repeated measures made at different times, for example among microfilarial counts from the same individual

at day 0 and day 90 was given a separate, ‘longitudinal’ correlation parameter ρ_2. The models were fitted to the data using generalized estimating equations implemented with the geepack [6] package for R [7].

Supplementary Tables

Supplementary Table A. Community Microfilarial Load of *Onchocerca volvulus* in 2004 [8], 2010 (this study, before introduction of biannual ivermectin treatment) and 2013 (this study, after 4 or 5 rounds of biannual ivermectin treatment) in 10 sentinel communities of the Neglected Tropical Disease Programme of the Ghana Health Service

Community	Community Microfilarial Load					
	Oct 2004[a]	Jul 2010 (95% CI[b])	% change Oct 2004 to Jul 2010	Mar 2013 (95% CI[b])	Jun 2013 (95% CI[b])	% change Jul 2010 to Mar/Jun 2013
Agborlekame 1	NA[c]	1.04 (0.56, 1.73)	NA	0.31 (0.11, 0.62)	NA	− 70%
Asubende	0.62	0.33 (0.13, 0.58)	− 47%	NA	0.17 (0.03, 0.38)	− 48%
Baaya	0.28	0.00 (0.00, 0.01)	NC[d]	NA	0.03 (0.00, 0.07)	NC[d]
Jagbenbendo	2.12	1.09 (0.74, 1.56)	− 52%	0.58 (0.33, 0.94)	NA	− 47%
Kyingakrom	2.85	0.32 (0.15, 0.57)	− 89%	NA	0.35 (0.09, 0.77)	+ 10%
New Longoro	1.42	0.20 (0.093, 0.33)	− 86%	0.01 (0.00, 0.04)	NA	− 95%
Ohiampe	0.21	0.091 (0.02, 0.19)	− 57%	NA	0.15 (0.01, 0.36)	+ 65%
Senyase	0.36	0.11 (0.03, 0.21)	− 69%	NA	0.04 (0.00, 0.12)	− 36%
Takumdo	NA	1.57 (1.01, 2.29)	NA	0.17 (0.03, 0.37)	NA	− 89%
Wiae	1.20	0.35 (0.19, 0.56)	− 71%	0.10 (0.03, 0.19)	NA	− 71%

[a] Estimates presented by Osei-Atweneboana et al. [8]; [b] confidence interval, estimated using the numerical bootstrap approach outlined in Supplementary Methods, *Community Microfilarial Load and Community Microfilarial Prevalence*; [c] NA = not available, community not studied at that time; [d] not calculated, only one individual positive for microfilariae in 2010 and 2013.

Supplementary Table B. Community Microfilarial Prevalence of *Onchocerca volvulus* in 2004 [8], 2010 (this study, before introduction of biannual ivermectin treatment) and 2013 (this study, after 4 or 5 rounds of biannual ivermectin treatment) in 10 sentinel communities of the Neglected Tropical Disease Programme of the Ghana Health Service

Community	Community Microfilarial Prevalence					
	Oct 2004[a]	Jul 2010 (95% CI[b])	% change Oct 2004 to Jul 2010	Mar 2013 (95% CI[b])	Jun 2013 (95% CI[b])	% change Jul 2010 to Mar/Jun 2013
Agborlekame 1	NA[c]	34.6% (24.6%, 47%)	NA	17.6% (6.1%, 29.3%)	NA	− 49%
Asubende	13.9%	20.1% (8.5%, 33%)	− 47%	NA	15.5% (2.9%, 29.7%)	− 22%
Baaya	8.7%	0.5% (0.0%, 1.6%)	NC[d]	NA	2.4% (0.0%, 5.8%)	NC[d]
Jagbenbendo	43.3%	38.3% (29.6%, 47.5%)	− 52%	25.7% (16.8%, 36.3%)	NA	− 33%
Kyingakrom	50.8%	12.6% (7.0%, 19.4%)	− 89%	NA	12.5% (4.5%, 22.9%)	− 0.8%
New Longoro	35.8%	9.8% (5.7%, 14.7%)	− 86%	2.5%[e] (0.0%, 6.3%)	NA	− 74%
Ohiampe	5.0%	4.2% (0.82%, 8.5%)	− 57%	NA	4.9% (1.2%, 10.0%)	+ 17%
Senyase	4.3%	9.1% (3.3%, 16%)	− 69%	NA	3.4% (0.0%, 8.8%)	− 63%
Takumdo	NA	37.8% (29.3%, 47.0%)	NA	6.6% (2.2%, 12.4%)	NA	− 83%
Wiae	38.5%	12.1% (7.7%, 16.7%)	− 71%	5.9% (1.7%, 10.0%)	NA	− 51%

[a] Estimates presented by Osei-Atweneboana et al. [8]; [b] confidence interval, estimated using the numerical bootstrap approach outlined in Supplementary Methods, *Community Microfilarial Load and Community Microfilarial Prevalence*; [c] NA = not available, community not studied at that time; [d] not calculated, only one individual positive for microfilariae in 2010 and 2013.

Supplementary Table C. Six-month *Onchocerca volvulus* microfilarial skin repopulation rates in 2004 [8] and 2010–2011 (this study, after introduction of biannual ivermectin treatment) in 10 sentinel communities of the Neglected Tropical Disease Programme of the Ghana Health Service

Community	Six-month microfilarial skin repopulation rates[a]		
	Nov 2004 – Apr 2005[b]	Jul 2010 – Jan 2011 (95% CI[c])	Jan 2011 – Jul 2011 (95% CI[c])
Agborlekame 1	NA[d]	50.1% (26.5%, 94.9%)	26.1% (10.6%, 64%)
Asubende	22.1%	53.8% (26%, 112%)	129% (48.9%, 250%)[f]
Baaya	12.3%	NR[e]	NR[e]
Jagbenbendo	36.3%	46.2% (28%, 76.2%)	17.9% (7.2%, 44.8%)
Kyingakrom	53.8%	69.6% (34%, 143%)	94.3% (40.8%, 218%)
New Longoro	22.5%	41.6% (16.4%, 105%)	74.3% (26.1%, 211%)
Ohiampe	16.2%	54.3% (31%, 94.9%)	42.2% (7.7%, 231%)
Senyase	16.0%	45.8% (17.6%, 120%)[f]	53.4% (12.6%, 227%)
Takumdo	NA[d]	49.4% (30.7%, 79.4%)	22.1% (10.5%, 46.6%)
Wiae	29.6%	44.4% (22%, 89.8%)[f]	25.2% (5.6%, 114%)

[a] the mean number of microfilariae (mf per skin snip, ss) per participant six months after a round of ivermectin treatment expressed as a percentage of the mean number of mf/ss per participant just before the preceding round of treatment; [b] estimates from data presented by Osei-Atweneboana et al. [8]; [c] confidence interval, estimated using the numerical bootstrap approach outlined in Supplementary Methods, *Community Microfilarial Load and Community Microfilarial Prevalence*; [d] NA = not available, community not studied at that time; [e] NR = not reported due to insufficient sample size and excessively large associated uncertainties; [f] upper bound truncated at 250%.

Supplementary Figures

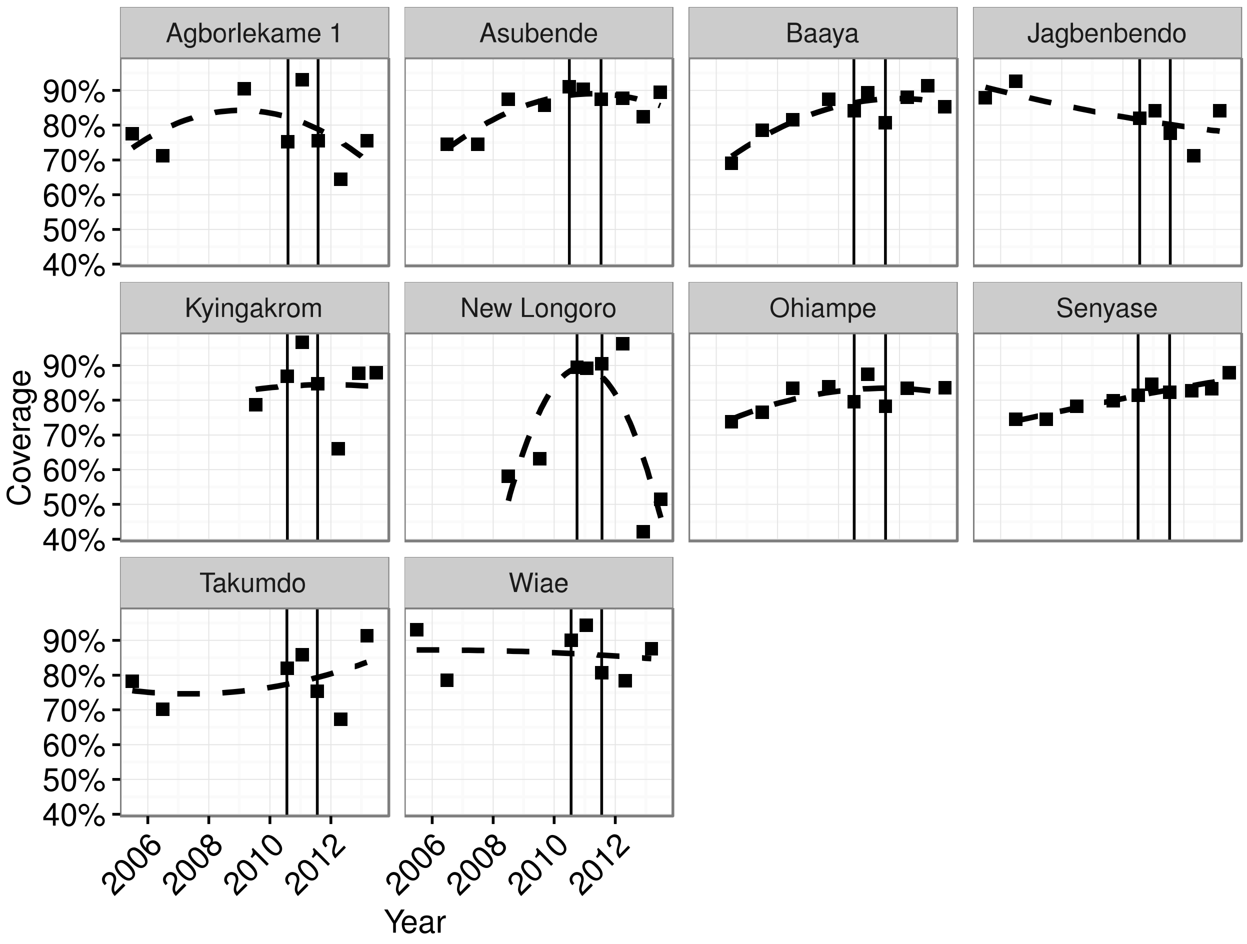

Supplementary Figure A. Trends in community coverage of ivermectin coverage in 10 Ghanaian communities. Data points represent the community coverage of ivermectin distribution reported by the Neglected Tropical Diseases Programme of the Ghana Health Services. Dotted lines are quadratic polynomial regression functions fitted by ordinary least squares to highlight the trends in the data. The horizontal lines on each panel represent the start and end of the component of the study (see Figure 2 in the main text) investigating two six-month periods of microfilarial repopulation in a longitudinal cohort through consecutive rounds of biannual treatment with ivermectin.

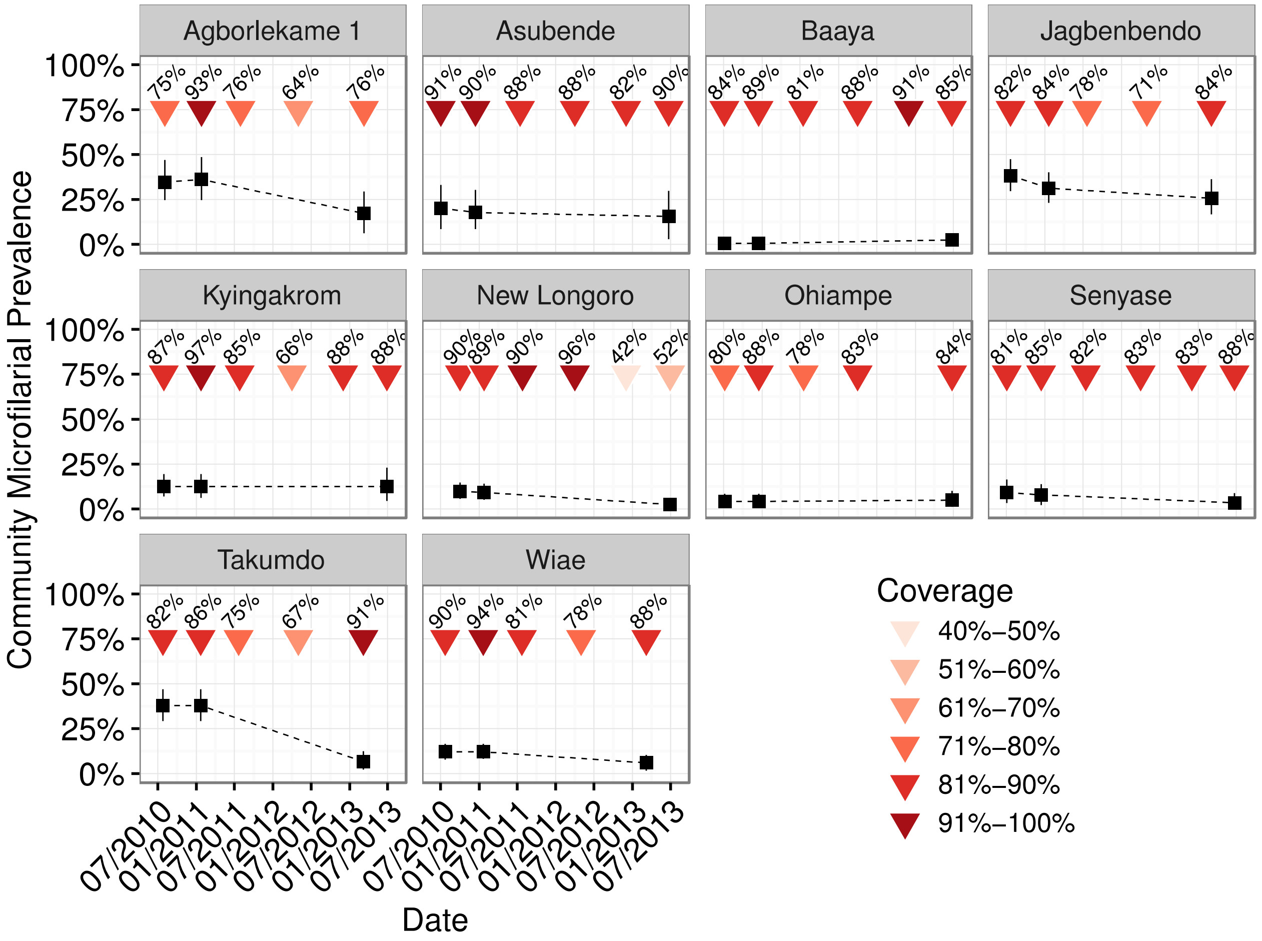

Supplementary Figure B. Trends in community microfilarial prevalence in 10 Ghanaian communities from the onset of a biannual ivermectin treatment strategy. Community Microfilarial Prevalence (CMFP) is defined as the prevalence of microfilariae in people aged 20 years and above where the presence of microfilariae is determined by the detection of microfilariae in either one of two (iliac) skin snips. Coloured arrows indicate dates when mass treatment with ivermectin was distributed, by either the authors or the Community Ivermectin Distributors. Ivermectin was administered directly after skin snipping on dates when microfilarial load was assessed. Data on the community therapeutic coverage of ivermectin were collated by the Ghana Health Service. Note that the six scheduled rounds of biannual ivermectin treatment were successfully delivered to only 5 (Asubende; Baaya; Kyingakrom; New Longoro and Senyase) out of the 10 communities; the others communities (Agborlekame 1; Jagbenbendo; Ohiampe; Takumdo, and Wiae) achieved five rounds of biannual treatment. Vertical error bars indicate 95% confidence intervals calculated using a non-parametric bootstrap technique (see Supplementary Methods, *Community Microfilaria Load and Community Microfilarial Prevalence*). Dotted lines join estimated values and are for presentation purposes

only. Triangles indicate times of ivermectin treatment and numbers above triangles indicate the therapeutic coverage in the whole community.

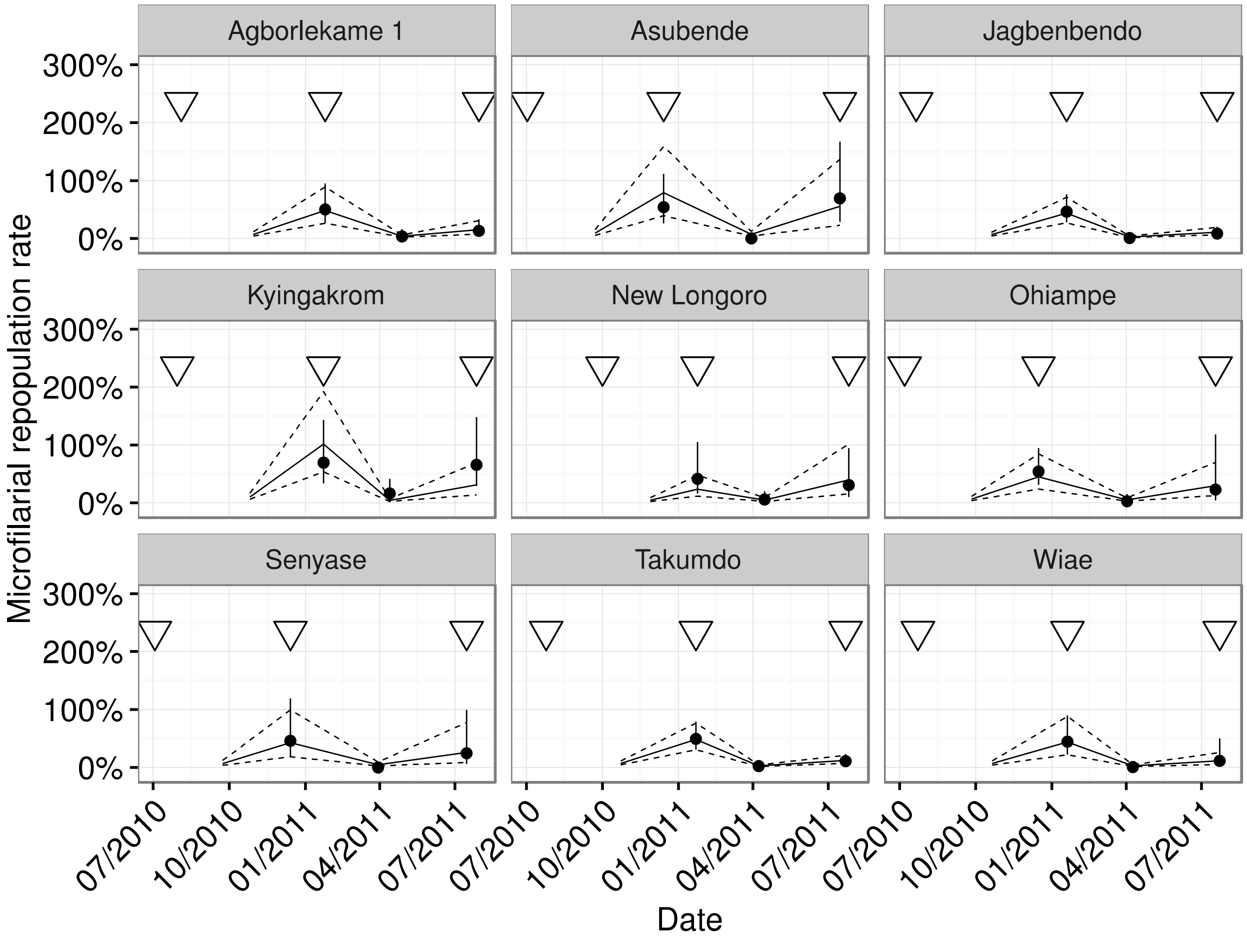

Supplementary Figure C. Microfilarial repopulation rates in 10 Ghanaian communities from the onset of a biannual ivermectin treatment strategy. Filled data points represent estimated mean microfilarial loads expressed as a percentage of the microfilarial load estimated just before the preceding round of ivermectin treatment. These estimates are derived from the marginal regression model that includes additive stratum adjustments for age group and sex, and interactive adjustments between sampling date and community (Model 1A; see Table 2 in the main text). Note that the formulation of this model means that repopulation rates are constant among demographic strata within a community, but variable among communities and between repopulation periods. Note also that rates of microfilarial repopulation from Baaya are not shown because only 1 participant was microfilaria-positive in this community (see Table 1 in the main text), leading

to very large associated estimates of uncertainty. The lines join estimates derived from the marginal regression model that includes time since the preceding round of ivermectin as a continuous covariate (Model 2; see Table 2 in the main text), permitting information from both repopulation periods to be combined into a single community-specific microfilarial repopulation rate, compared in Figure 8. The key assumption of Model 2 is that numbers of microfilariae between 3 and 6 months increase approximately log-linearly. The concordance between the lines and data points indicates the validity of this assumption. The solid vertical lines indicate 95% confidence bounds associated with estimates from Model 1A, and the dotted lines join 95% confidence bounds associated with estimated from Model 2, both calculated using robust sandwich estimators of coefficient standard errors (see Supplementary Methods, *Marginal regression models*). Triangles indicate times of ivermectin treatment.

Supplementary References

1. Moreau JP, Prost A, Prod'hon J. [An attempt to normalize the methodology of clinico-parasitologic surveys of onchocerciasis in West-Africa (author's transl)]. Med Trop (Mars) **1978;** 38:43–51 [Article in French].

2. Remme J, Ba O, Dadzie KY, Karam M. A force-of-infection model for onchocerciasis and its applications in the epidemiological evaluation of the Onchocerciasis Control Programme in the Volta River basin area. Bull World Health Organ **1986;** 64:667–81.

3. Davison AC, Hinkley DV. Bootstrap Methods and Their Application. Cambridge: Cambridge University Press; **1997.**

4. Hanley JA, Negassa A, Edwardes MD, Forrestor JE. Statistical analysis of correlated data using generalized estimating equations: an orientation. Am J Epidemiol **2003;** 157:364–75.

5. Gardon J, Boussinesq B, Kamgno J, Gardon-Wendel N, Demanga-Ngangue, Duke BOL. Effects of standard and high doses of ivermectin on adult worms of *Onchocerca volvulus*: a randomised controlled trial. Lancet **2002;** 360:203–10.

6. Halekoh U, Højsgaard S, Yan J. The R package geepack for generalized estimating equations. J Stat Softw **2006;** 15:1–11.

7. R Core Team. R: a language and environment for statistical computing. Vienna: R Foundation for Statistical Computing; **2015.**

8. Osei-Atweneboana MY, Eng JKL, Boakye DA, Gyapong JO, Prichard RK. Prevalence and intensity of *Onchocerca volvulus* infection and efficacy of ivermectin in endemic communities in Ghana: a two-phase epidemiological study. Lancet **2007;** 369:2021–29.
